# T56. AN EXPLORATORY ANALYSIS CONVERTING SCORES BETWEEN THE PANSS AND BNSS

**DOI:** 10.1093/schbul/sby016.332

**Published:** 2018-04-01

**Authors:** Alan Kott, David Daniel

**Affiliations:** Bracket Global, LLC

## Abstract

**Background:**

The Brief Negative Symptom Scale is a relatively new instrument designed specifically to measure the negative symptoms in schizophrenia. Recently more clinical trials include the BNSS scale as a secondary or exploratory outcome, typically along with the PANSS. In the current analysis we aimed at establishing the equations that would allow conversion between the BNSS scale total score and the PANSS negative subscale and PANSS negative factors score as well as conversion equations between the expressive deficits and avolition/apathy factors of the scales. (Kirkpatrick, 2011; Strauss, 2012)

**Methods:**

Data from 518 schizophrenia clinical trials subjects with both PANSS and BNSS data available were used. Regression analyses predicting the BNSS total score with the PANSS negative subscale score, and the BNSS total score with the PANSS Negative factor (NFS) score were performed on data from all subjects. Regression analyses predicting the BNSS avolition/apathy factor (items 1, 2, 3, 5, 6, 7, and 8) with the PANSS avolition/apathy factor (items N2, N4 and G16) and the BNSS expressive deficits factor (items 4, 9, 10, 11, 12, and 13)with the expressive deficits factor (items N1, N3, N6, G5, G7, and G13)of the PANSS were performed on a sample of 318 subjects with individual BNSS item scores available. In addition to estimating the equations we as well calculated the Pearson’s correlations between the scales.

**Results:**

The PANSS and BNSS avolition/apathy factors were highly correlated (r=0.70) as were the expressive deficit factors r=0.83). The following equations predicting the BNSS total score were obtained from regression analyses performed on 2,560 data points:

BNSS_total = -11.64 + 2.10*PANSS_negative_subscale

BNSS_total = -9.26 + 2.11*PANSS_NFS

The following equations predicting the BNSS factor scores from the PANSS factor scores were obtained from regression analyses performed on 1,634 data points:

BNSS_avolition/apathy = -2.40 + 2.38 * PANSS_avolition/apathy

BNSS_expressive_deficit_factor = -4.21 + 1.27 * PANSS_expressive_deficit_factor

**Discussion:**

The BNSS differs from the PANSS negative factor because it addresses all five currently recognized domains of negative symptoms including anhedonia and attempts to differentiate anticipatory from consummatory states. In our analysis we have replicated the strong correlation between the BNSS total score and PANSS negative subscale and newly identified strong correlations between the BNSS total score and NFS as well as strong correlations between the avolotion/apathy and expressive deficit factors of the BNSS and the PANSS scales. (Kirkpatrick, 2011)The provided equations offer a useful tool allowing researchers and clinicians to easily convert the data between the instruments for reasons such as pooling data from multiple trials using one of the instruments, to allow interpretation of results within the context of previously conducted research, etc. but as well offer a framework for risk based monitoring to identify data deviating from the expected relationship and allow for a targeted exploration of the causes for such a disagreement. The data used for analysis included not only subjects with predominantly negative symptoms but as well acutely psychotic subjects as well as subjects in stable conditions allowing therefore to generalize the results across the majority of schizophrenic subjects. This post-hoc analysis is exploratory. We plan to further explore the potential utility of equations addressing the relationships among schizophrenia measures of symptom severity in an iterative manner with larger datasets.

